# A Survey on the Awareness of Epilepsy Treatments in Community Pharmacies

**DOI:** 10.7759/cureus.79249

**Published:** 2025-02-18

**Authors:** Hiroshi Yoshikawa, Shuichiro Neshige, Kota Kagawa, Shohei Sato, Ryo Nishigakiuchi, Kenji Fujii, Hirofumi Maruyama, Koji Iida, Hiroaki Matsuo

**Affiliations:** 1 Department of Pharmaceutical Services, Hiroshima University Hospital, Hiroshima, JPN; 2 Department of Clinical Neuroscience and Therapeutics, Hiroshima University Graduate School of Biomedical and Health Sciences, Hiroshima, JPN; 3 Department of Neurosurgery, Hiroshima University Graduate School of Biomedical and Health Sciences, Hiroshima, JPN; 4 Epilepsy Center, Hiroshima University Hospital, Hiroshima, JPN

**Keywords:** antiseizure medications, drug-drug interactions, information sharing, lower seizure thresholds, pharmacy, switching to generic drugs

## Abstract

Background: For effective epileptic seizure management, pharmacies need to provide physicians with information regarding drug-drug interactions, drugs that lower seizure thresholds, and switching to generic drugs. The aim of the study was to clarify the actual situation of information provision from pharmacies to physicians and investigate awareness of epilepsy treatment in community pharmacies.

Methods: A survey targeting all pharmacies (1,512 pharmacies) belonging to the Hiroshima Prefectural Pharmacists Association was conducted from October 7, 2023, to November 30, 2023. The survey investigated the dispensing status of antiseizure medication (ASM) prescriptions and the feedback rate to physicians regarding drug interactions, seizure-threshold-lowering drugs, and switching to generics. We also evaluated the relationship between the frequency of patient care, the status of information provision, and switching to generic medications.

Results: Of the 197 pharmacies that responded, 181 (92%) had experience with ASM prescriptions. Ninety-five pharmacies (48%) provided feedback to physicians on interactions with newly prescribed ASMs. Precautions for interactions between new drugs and ongoing ASMs were addressed in 98 pharmacies (50%). Additionally, 86 pharmacies (44%) provided feedback on new drugs that lower the seizure threshold. Generic switching occurred in 143 pharmacies (73%). Information for physicians was provided via medicine notebooks in 160 cases (81%), while preconfirmation was conducted in only 10 cases (5%). Pharmacies providing patient care at least once a week were more likely to provide information about newly prescribed concomitant medications (p = 0.04), but no significant difference was found in other cases. They were also more likely to switch to generic drugs (p < 0.01), but there was no difference in providing information to physicians before confirmation.

Conclusions: Feedback rates to physicians regarding interactions requiring precautions and threshold-lowering drugs were 50% or less, and prior confirmation when switching to generic drugs was rare. Further promotion of hospital-pharmacy information sharing is needed for more appropriate seizure management in patients with epilepsy.

## Introduction

Antiseizure medications (ASMs) are essential for maintaining reliable suppression of seizures while ensuring safety during their use. Accurate dosage adjustment of ASMs is necessary, and consideration of drug interactions with concomitant medications is critical for treatment planning [1,2]. The use of medications that lower seizure thresholds, such as first-generation antihistamines, must also be carefully considered [1]. However, patients with epilepsy may receive prescriptions from multiple healthcare facilities, potentially leading to such problems. Furthermore, driven by national policies in Japan aimed at reducing healthcare costs and patient burdens, there has been an emphasis on switching to generic drugs across all drug classes. However, the Japanese Epilepsy Society and the Japanese Society of Pediatric Neurology have issued a warning about switching to generic ASMs because many ASMs have a narrow therapeutic range, there are concerns about seizure recurrence and side effects with small changes, and treatment equivalence has not been verified, which may lead to loss of seizure control [1]. Community pharmacists must inform prescribing physicians about switching medications to ensure patient safety. Effective communication between pharmacies and healthcare facilities is essential to manage concomitant medications and generic drug substitutions. In a report examining physician-pharmacist information-sharing tools for drug profiles and drug interaction screening, most physicians prefer to use pharmacists as a source of information [3]. However, there are few reports on the status of information sharing between pharmacies and physicians. Understanding how pharmacists from different pharmacies handle prescriptions for epilepsy treatment is vital for improving care standards. Therefore, a broad survey is necessary to assess the current practices comprehensively. This study aimed to conduct a survey of community pharmacies in Hiroshima Prefecture to determine the extent to which they provide information to physicians regarding drug interactions, medications that lower the seizure threshold, and switching to generic drugs as part of the management of patients with epilepsy.

## Materials and methods

This study was a survey of pharmacies in Hiroshima Prefecture. The survey period was from October 7, 2023, to November 30, 2023, and included 1,512 pharmacies belonging to the Hiroshima Pharmaceutical Association. Hiroshima Prefecture has a population of approximately 2.7 million (as of February 2024), ranking 12th among the 47 prefectures in Japan. The Hiroshima Pharmaceutical Association encompasses approximately 95% of the pharmacies in the prefecture, providing a representative overview of the status of pharmacists’ interventions with ASMs in this region. This study was approved by the Ethics Committee of Hiroshima University Hospital (approval number: E2023-0109, dated September 1, 2023).

Survey and data source

The survey form was distributed to the managing pharmacists of all the target pharmacies via fax and mail. Additionally, it was posted on the Hiroshima Pharmaceutical Association's website. Both the paper form and website included a QR code linking to Microsoft Forms (Microsoft, Redmond, WA) for the responses. Reminders were faxed, mailed, and posted on the website once to increase the response rate. Pharmacy names were collected as information to avoid duplication. In the case of duplicates, the latest response was adopted. The response to the survey constituted consent to participate in this study. Each managing pharmacist responded on their own, with one response per pharmacy. Pharmacies that did not fill prescriptions for epilepsy patients were also included in the survey and analysis. Responses were collected either online through Microsoft Forms or via fax.

Survey content and assessment

This study investigated the following aspects: 1) the status of prescription reception for ASMs, 2) drug interactions involving ASMs, 3) handling of medications that lower the seizure threshold, and 4) switching to generic ASMs. ASM interactions were defined as those that affect the therapeutic effect of ASMs, such as changes in their blood concentration, while medications that lower the seizure threshold were defined as those that alter the pathophysiology of epilepsy, such as antihistamines. The survey contents are presented in Appendix 1.

Status of prescription reception for ASMs

We surveyed pharmacists regarding the number of ASM prescriptions received and processed and the frequency of patient care, including dispensing and medication instruction for patients with epilepsy (every day, every two to three days, once a week, every two weeks, and once a month or less).

Drug interactions with ASMs and handling of drugs lowering the seizure threshold

To understand how pharmacists determine ASM interactions beyond using information on package inserts, we surveyed the sources of information they used. Additionally, to clarify how pharmacies respond and provide information to prescribing physicians when there are interactions that require precaution or there is a drug lowering the seizure threshold, we investigated their main actions in three scenarios: 1) when a new ASM prescription has an interaction with a patient’s regular medications, which requires precautions, 2) when a new medication is prescribed to a patient already on ASMs, which has an interaction requiring precautions, and 3) when medications that lower the seizure threshold were prescribed. The survey included inquiries on patient counseling, inquiries, and information provision to prescribers of concomitant medications and ASMs and subsequent symptom monitoring. Moreover, we collected open-ended feedback on the tools pharmacies would consider useful for managing interactions and medications that lower the seizure threshold.

Switching to generic ASMs

Related academic societies have made recommendations regarding switching from branded ASMs to generic drugs. To ascertain the current practices, it was essential to investigate whether pharmacies had experienced switching from branded ASMs (for epilepsy treatment) to generics. The information gathered included the circumstances under which such switches occurred (new prescription, confirmed by the prescribing physician, patient request, or if the patient's original medication was already generic), the methods used by pharmacies to inform prescribing physicians, and any issues of worsening in seizure control arising from patient interviews after the switch to generics.

Relationship between frequency of patient care, information provision, and switching to generic medications

To examine the impact of the frequency of patient care on information provision and switching to generic medications, we examined the relationship between the frequency of patient care, the status of information provision, switching to generic medications, and circumstances under which such changes occur. For the purposes of the analysis, pharmacies that provided care to patients with epilepsy at least once a week were categorized as the “frequent” group, whereas all other pharmacies were categorized as “others.”

Statistical analysis

Survey responses were analyzed as a percentage. Missing data were included in the population for analysis. The relationship between the frequency of patient care, information provision, and switching to generic medications was analyzed using group comparison with the chi-square test (χ²) or Fisher's exact probability test. The analysis was performed using JMP PRO 17 (SAS Institute Inc., Cary, NC), with the significance level set at 5%.

## Results

Overview of responding pharmacies

Responses were obtained from 197 pharmacies for a response rate of 13% (Table 1). Most of the responding pharmacies frequently received prescriptions from hospitals. The median number of pharmacists per pharmacy was three (interquartile range: 2-4.3).

**Table 1:** Overview of pharmacies responding to the survey

Questionnaire	n
Number of responding pharmacies	197
Primary prescription source (multiple choices possible)	
Hospitals with epilepsy care	79
Hospitals without epilepsy care	50
Neurology clinics	22
Neurosurgery clinics	36
Pediatric clinics	38
Psychiatry clinics	39
Pharmacies located near hospitals with epilepsy specialists	9
Pharmacies located near epilepsy support network hospitals	12
Prescription demand for patients with epilepsy	
Yes	181
No	16
Frequency of care for patients with epilepsy	
Every day	14
Every two to three days	20
Once a week	19
Every two weeks	35
Once a month or less	106
Methods for checking drug interactions (multiple choices possible)	
Package inserts/documents that complement the contents of package insert	179
Automatic checking systems	118
Drug interaction screening tools	4

Status of prescription reception for ASMs

Of the responding pharmacies, 181 (92%) handled prescriptions for patients with epilepsy. The most common frequency of care was monthly or less, reported by 106 pharmacies (54%). Pharmacies attending to patients with epilepsy for at least one week numbered 53 (27%). The primary prescription sources were hospitals treating patients with epilepsy, which accounted for 79 pharmacies (40%). Among these, nine pharmacies (5%) were located near hospitals with epilepsy specialists, and 12 (6%) were situated near hospitals within the epilepsy support network that provided specialized epilepsy care and collaborated with hospitals with epilepsy specialists. Pharmacies attending to patients with epilepsy at least once per week tended to obtain prescriptions from such hospitals and were located near epilepsy specialists or network hospitals (Table 1, Appendix 2).

ASM drug interactions and handling of drugs that lower the seizure threshold

**Figure 1:**
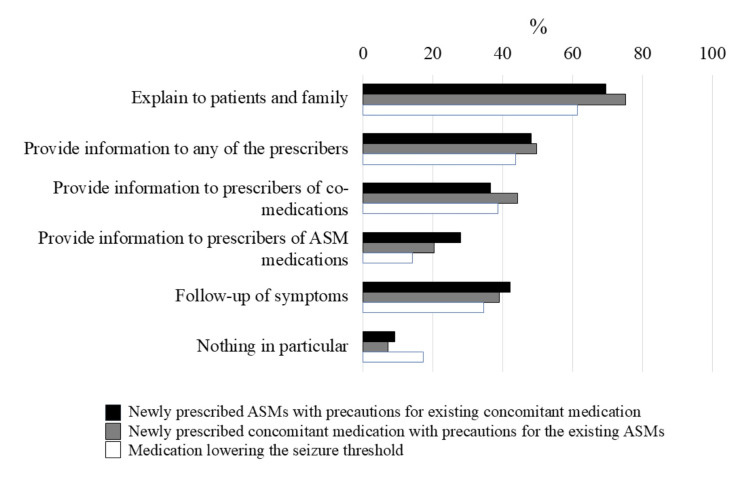
Pharmacy responses to drug interactions and seizure-threshold-lowering medications ASM: antiseizure medication

The most used tools for checking drug interactions in pharmacies were package inserts and documents that complemented the contents of package inserts, which were utilized by 179 (91%) of the respondents (Table 1). When a new ASM prescription interacts with a patient’s regular medications that require precautions, when a new medication is prescribed for a patient already taking ASMs that has an interaction requiring precautions, and when medications that lower the seizure threshold are prescribed, the most common response was to explain to the patient or their caregiver family. Ninety-five pharmacies (48%) provided information to any of the prescribers about new ASM prescriptions involving interactions with current medications, 98 pharmacies (50%) informed about new medications that interact with ongoing ASMs requiring precautions, and 86 pharmacies (44%) addressed new drugs that lower seizure thresholds. The number of pharmacies providing information to prescribers of concomitant medications was 72 (37%), 87 (44%), and 76 (39%), respectively, while the number to ASMs prescribers were 55 (28%), 40 (20%), and 28 (14%), respectively (Figure 1). The desired tools for checking drug interactions and drugs that lower the seizure threshold included electronic access to medication histories, a list of drug interactions for physician inquiry, and interaction summaries (Appendixes 3, 4).

Switching to generic ASMs

Switching from branded to generic ASMs was reported by 143 pharmacies (73%). The reasons included patient request 145 (74%), original generic medication 113 (57%), new prescriptions 66 (34%), and confirmation by a physician 29 (15%). When providing information to prescribing physicians, 160 pharmacies (81%) primarily used medicine notebooks, and only 10 pharmacies (5%) reported confirming the switch to a physician before dispensing a generic medicine. Moreover, 14 pharmacies (7.7%) experienced worsening epilepsy control after switching to a generic drug (Table 2).

**Table 2:** Switching to generic drugs ASMs: antiseizure medications

Questionnaire	n
Switching to generic ASMs	
Yes	143
No, we have not changed the ASMs (for epilepsy purposes)	50
Situation of switching generic drugs (multiple choices possible)	
New prescription	66
When confirmed by the prescribing physician	29
Patient's request	145
Patient was originally using generic medication	113
Other	6
How do you inform the prescribing physician after switching to generic drugs? (multiple choices possible)	
Medicine notebook	160
Report on medication information and related details from the pharmacy	16
Phone contact (preconfirmation)	10
Others	10
Experienced poor control or similar issues after switching to generic drugs	
Yes	14
No	168

Relationship between frequency of patient care, information provision, and switching to generic medications

Pharmacies that attended to patients with epilepsy at least once a week (the frequent group) had higher information provision rates for newly prescribed concomitant medications (p = 0.04). No substantial differences were observed between the groups for information provision in other scenarios. The frequent group was significantly more likely to switch patients to generic medications (p < 0.01). The frequent group also favored switching to generics, significantly so in the case of new prescriptions (p < 0.01). No significant differences between the groups were observed when switching to generic medicines under other conditions such as patient requests (Table 3).

**Table 3:** Relationship between frequency of care and response to information provision, as well as switching to generic drugs for patients with epilepsy ^a^Chi-square test (χ²) ASMs: antiseizure medications

Addition or switch of medication	Information recipient	Care for patients with epilepsy		p value^a^	Chi-square value
		Frequent group (n = 53), n (%)	Others (n = 141), n (%)		
Precautions with new ASMs and concomitant medications	Any prescribers	28 (53)	66 (47)	0.45	0.559
	Concomitant medication prescribers	20 (38）	51 (36)	0.84	0.041
	ASM prescribers	16 (30)	39 (28)	0.73	0.121
Precautions with ASMs and new concomitant medications	Any prescribers	33 (62)	65 (46)	0.04	4.027
	Concomitant medications prescribers	30 (57)	57 (40)	0.04	4.076
	ASM prescribers	13 (25)	27 (19)	0.41	0.681
Newly prescribed medication lowering seizure threshold	Any prescribers	18 (34)	68 (48)	0.07	3.176
	Prescribers of drugs that lower the seizure threshold	16 (30)	60 (43)	0.11	2.471
	ASM prescribers	8 (15)	20 (14)	0.87	0.026
Switch to generic drugs	-	48 (92.3)	94 (67.6)	<0.01	12.087
Situation of switching generic drugs	New prescription	26 (49.1)	40 (28.4)	<0.01	7.345
	When confirmed by the prescribing physician	10 (18.9)	20 (14.2)	0.42	0.646
	Patient's request	43 (81.1)	100 (70.9)	0.15	2.072
	Patient was originally using generic medication	35 (66.0)	77 (54.6)	0.15	2.062

## Discussion

The study revealed that pharmacies do not provide sufficient information to physicians about problematic concomitant medications (interactions and drugs that lower thresholds) and changes to generic drugs. Pharmacists commonly explain drug interactions and seizure-threshold-lowering medications to patients and their families. However, they often fail to provide adequate information to any of the prescribers; the rates for newly prescribed ASMs, concomitant medications requiring precautions with ASMs, and seizure-threshold-lowering drugs were 48%, 50%, and 44%, respectively. Similar information was provided to ASM prescribers and concomitant drug prescribers: 28%, 20%, and 14% for ASM prescribers, and 37%, 44%, and 39% for concomitant drug prescribers, respectively; all values are less than 50%. This suggests a lower tendency to provide information to ASM-prescribing physicians. There is a need for standardized tools, such as medication inquiry lists and interaction summaries, to improve consistency among pharmacies.

Switching to generic ASMs was reported by 74% of the pharmacies. The most common method for informing prescribing physicians about these changes was through the patient’s medicine notebook, which was used by 81% of pharmacies. In contrast, preconfirmation with the prescribing physician was made by only approximately 5%. This infers that many physicians may not be able to discuss the use of generic drugs with their patients. Furthermore, 7.7% of pharmacies experienced a worsening of control after switching to generic drugs. This is considered an unacceptable number considering the impact on daily life, such as driving a car. On the other hand, epilepsy can change seizure control over the natural course of the disease, and further research would be needed to determine a causal relationship with the change to generics.

Analysis of the relationship between the frequency of patient care and information provision or switching to generics revealed several trends. Pharmacies with higher frequencies of patient care (at least once a week) were significantly more likely to provide information to prescribing physicians regarding concomitant medications when a new medication with an ASM interaction requiring precautions was added. However, other types of information provision were not related to the frequency of patient care. Pharmacies providing more frequent patient care were also significantly more likely to switch to generic medications. Although they were more likely to switch at the start of the new prescription, among the groups, the switch was not consistently confirmed with the physician prior to dispensing the drug to the patient. Pharmacists should prioritize proactive communication with ASM-prescribing physicians and patients before switching to generic ASMs.

This survey examines how pharmacies manage the care for patients with epilepsy in Japan. The survey was conducted in 1,512 pharmacies, many of which are pharmacies in the region, and is considered to reflect the attitudes of pharmacies toward epilepsy drugs in the region. This survey revealed the actual status of information provided by pharmacies to physicians about interactions, drugs that lower the epilepsy threshold, and switching generic medication to treat epilepsy. Newer ASMs such as levetiracetam and lamotrigine have fewer drug interactions than traditional ASMs such as phenytoin, phenobarbital, carbamazepine, and valproic acid (VPA) [4]. However, traditional ASMs are commonly prescribed [5], leading to interactions with other medications [2,6]. Recognizing drugs that lower the seizure threshold is crucial according to Japanese and international guidelines and advising caution only when necessary [1]. Centralized prescriptions in pharmacies may help resolve these complex issues; however, there is no quantitative evaluation of the degree to which interactions and reduced seizure thresholds impact ASM effectiveness and epilepsy control, and the lack of standards for providing information to physicians may make it difficult for pharmacies to respond. Regarding generic ASM switches, Japanese guidelines caution against changing medications in patients with well-controlled seizures without consent from both providers and patients [1]. There have been reports of diminished efficacy following switching to generic lamotrigine, which improved upon reversion to the original brand [6]. However, distribution problems may prevent the delivery of the same drug being used by the patient. A report by Matsunuma et al. on the response of pharmacies to VPA supply problems revealed that many pharmacies were changing from brand to generic or from generic to brand in supply problems [7]. Although there were no reports of poor control in this report, 80% of pharmacies did not collaborate with physicians, suggesting a lack of collaboration like this study. The approval criteria for generic drugs stipulate an 80%-125% bioequivalence range. While the 90% confidence interval requirement ensures that the actual differences in Cmax (maximum blood concentration) and area under the concentration-time curve between generic and brand-name drugs are typically smaller than the acceptance range [8], concerns have been raised regarding the appropriateness of this range for ASMs. Given their pharmacokinetic variability and the potential clinical impact of small fluctuations in drug levels, some experts suggest that ASMs should be designated as narrow therapeutic index drugs to ensure stricter bioequivalence requirements [9]. Despite these cautions, most pharmacies switch to generic drugs, often driven by patient requests, with minimal confirmation from prescribing physicians, even in high-frequency epilepsy care settings. Preswitch communication with physicians remained low (5%), highlighting gaps in healthcare provider awareness and communication. The Hong Kong guidelines [10] emphasize communication between physicians and patients regarding generic substitutions, highlighting concerns about changes in medication appearance affecting adherence.

This study has several limitations. First, the survey response rate was low, which may have limited the generalizability of the survey. The low response rate may be due to the large number of pharmacies (1,512), which meant that the survey responses were requested by email or fax and were not well publicized and that it was difficult for pharmacies that care less about epilepsy patients to respond to the survey. Some of the questions were of an open-ended nature, which could also be a reason for the low response rate. Furthermore, the number of pharmacies caring for epilepsy patients may have been small. It is possible that the response rate would have been higher if only those pharmacies that care for epilepsy patients were asked to respond. However, responses were obtained from a wide range of pharmacies based on the demand for prescriptions, number of pharmacists, and frequency of care for patients with epilepsy, suggesting minimal bias in the functional and situational characteristics of the responding pharmacies. Second, this study was unable to extract the pharmacies dispensing medication to epilepsy patients and predict the survey response rate, so we sent the questionnaire to all member pharmacies of the Hiroshima Pharmaceutical Association. The fact that we were ultimately unable to secure a sufficient sample size is considered to be a limitation of this study. Third, this survey was directed toward managing pharmacists at pharmacies, where responses may not necessarily be uniform within the pharmacy, potentially leading to differences in approaches among pharmacists, even within the same pharmacy. Fourth, a limitation of surveys is that responses are self-reported, making objective verification difficult. Further investigation into the more detailed pharmacy behavior is considered necessary.

Our interpretation of this study is that one of the reasons for the lack of information sharing is the lack of standardized criteria for information provision, which suggests variability in responses among pharmacies. Additionally, a report examining the effectiveness of interventions, including patient-pharmacist consultations, care plan development, regular check-ins, and care coordination in pharmacies, indicated that the lack of opportunities to intervene with epilepsy patients in pharmacies constitutes a barrier [11]. However, even in pharmacies with a high frequency of patient care, information sharing on interactions and switching to generics was shown to be inadequate. These results suggest that pharmacies with large numbers of epilepsy patients would benefit from additional education and training on ASMs, including aspects such as additional monitoring requirements when switching patients to generic medicines.

Various surveys have been conducted to assess pharmacists' knowledge of epilepsy. Canadian pharmacists' knowledge of epilepsy is influenced by hospital experience, years since graduation, neurological exposure, and the need for educational tools [12]. German reports highlight insufficient seizure management knowledge and advocate for regular training [13]. A Palestinian study showed that pharmacists who received training on epilepsy and ASMs scored higher for knowledge of the pharmacotherapy of epilepsy than those who had not received training, indicating significant knowledge gaps [14]. Our findings suggest limited sharing of information with physicians and disparities in experiences of managing patients with epilepsy, emphasizing the need for information sharing in epilepsy care.

## Conclusions

This study highlighted that information sharing with physicians about potential interactions with concomitant medications and drugs that may lower the threshold for epilepsy was inadequate at 50% or less. Moreover, it was evident that although many pharmacies switch patients to generic drugs, there is inadequate prior communication with physicians prescribing ASMs. These findings suggest the need for increased information sharing with pharmacies concerning the management of patients with epilepsy.

## Appendices

Appendix 1

**Table 4:** Questionnaire content ASMs: antiseizure medications; SSRIs: selective serotonin reuptake inhibitors

Questionnaire
The status of prescription reception
Have you handled patients with epilepsy?
・Yes
・No
Frequency of dealing with patients with epilepsy
・Every day
・Every 2-3 days
・Once a week
・Every 2 weeks
・Once a month or less
ASM interactions
What do you refer to when checking for interactions? (multiple choices allowed)
・Package inserts, documents that complement the contents of the package insert
・Automatic system checks
・Interaction search tools (e.g., SAFE-DI, Lexicomp)
Regarding the case where a new ASMs is prescribed (if ASMs and the drug precautions when used concomitantly are prescribed by different medical institutions): If a drug that the patient is currently taking is labeled "precautions" with the newly started ASMs, how do you respond? (Assuming the impact is on ASMs) (Multiple answers allowed)
・Explain to the patient or family
・Contact the prescribing physician for precautionary medications
・Provide information to the prescribing physician about precautionary medications
・Contact the prescribing physician of ASMs
・Provide information to the prescribing physician of ASMs
・Follow up on the patient's subsequent symptoms
・Do nothing in particular
How do you respond when a new precautionary medicine is prescribed to a patient taking an ASMs? (Assuming the impact is on the ASMs) (Multiple answers allowed)
・Explain to the patient or family
・Contact the prescribing physician for precautionary medications
・Provide information to the prescribing physician about precautionary medications
・Contact the prescribing physician of ASMs
・Provide information to the prescribing physician of ASMs
・Follow up on the patient's subsequent symptoms
・Do nothing in particular
What kind of tools would be useful for checking drug interactions?
-
Drugs that lower the epilepsy threshold
Examples of drugs that lower the epilepsy threshold [1]
・Antidepressants (e.g., imipramine, amitriptyline, mild SSRIs)
・Antipsychotics (e.g., chlorpromazine, thioridazine)
・Bronchodilators (e.g., aminophylline, theophylline)
・Antimicrobials (e.g., quinolones)
・Analgesics (e.g., fentanyl)
・Antitumor drugs (vincristine, methotrexate)
・Muscle relaxants (e.g., baclofen)
・Antihistamines (especially first generation)
How do you respond when a new drug that lowers the epilepsy threshold is prescribed to a patient with epilepsy? (Multiple answers allowed)
・Explain to the patient or family
・Contact the prescribing physician of the drug that lowers the epilepsy threshold
・Provide information to the prescribing physician of the drug that lowers the epilepsy threshold
・Contact the prescribing physician of ASMs
・Provide information to the prescribing physician of ASMs
・Follow up on the patient's subsequent symptoms
・Do nothing in particular
What kind of tools would be useful for checking drugs that lower the epilepsy threshold?
-
Switch to generic ASMs
Have you ever switched ASMs to generics?
・Yes
・No (I have not changed ASMs for epilepsy purposes)
In what situations do you switch generic drugs? (Multiple answers allowed)
・New prescription
・When confirmed by the prescribing physician
・Patient request
・When the patient’s original medication was a generic drug
・other
When changing to generic drugs, how do you provide information to the prescribing physician? (Multiple answers possible)
・Medicine notebook
・Report on medication information and related details from the pharmacy
・Phone contact (pre-confirmation)
・other
Have you experienced poor control or similar issues after switching to generic drugs?
・Yes
・No

Appendix 2

**Table 5:** Relationship between prescribing institutions and frequency of care for patients with epilepsy ^a^Fisher's exact probability test

Pharmacy	Care for patients with epilepsy		p value^a^	Chi-square value
	Frequent group (n = 53), n (%)	Others (n = 141), n (%)		
Primary prescription sources were hospitals with patients with epilepsy	35 (66）	44 (31)	<0.01	19.361
Pharmacies located near hospitals with epilepsy specialists	5 (9)	4 (3)	0.06	3.790
Pharmacies located near epilepsy support network hospitals	7 (13)	5 (4)	0.02	6.196

Appendix 3

**Table 6:** Types of tools useful for checking drug interactions

Tool	n
Patient medication history, test results, etc., electronically accessible	9
Summary of the extent of drug interaction impacts	9
List of medications requiring consultation with physicians	9
Clinical information (including information in medical records)	2
Others	
AI-powered search tools	1
Tools that provide insights into the pathophysiology of epilepsy	1

Appendix 4

**Table 7:** Types of tools useful for checking drugs that lower the seizure threshold

Tool	n
List of medications requiring consultation with physicians	7
Summary of the extent of impact when medications are used concomitantly	3
Patient medication history, test results, etc., accessible electronically	2
Others	
Clinical information (including information in medical records)	1
AI-powered search tools	1
Tools that provide insights into the pathophysiology of epilepsy	1
Searching tool for drugs that lower seizure thresholds in pharmacy systems	1
